# Risk Assessment of Fluoride Intake from Tea in the Republic of Ireland and its Implications for Public Health and Water Fluoridation

**DOI:** 10.3390/ijerph13030259

**Published:** 2016-02-26

**Authors:** Declan T. Waugh, William Potter, Hardy Limeback, Michael Godfrey

**Affiliations:** 1 EnviroManagement Services, 11 Riverview, Dohertys Rd, Bandon, Co. Cork P72 YF10, Ireland; 2 Department of Chemistry and Biochemistry, KEH M2225, University of Tulsa, Tulsa, OK 74104-3189, USA; william-potter@utulsa.edu; 3 Faculty of Dentistry, University of Toronto, 124 Edward Street, Toronto, ON M5G 1G6, Canada; hardy.limeback@gmail.com; 4 Bay of Plenty Environmental Health, 1416A Cameron Road, Tauranga 3012, New Zealand; mike@godfreymedical.nz

**Keywords:** tea, fluoride, risk assessment, Public Health Safety, skeletal fluorosis, chronic pain, endocrine disorders, neurotoxicity, water fluoridation, food safety

## Abstract

The Republic of Ireland (RoI) is the only European Country with a mandatory national legislation requiring artificial fluoridation of drinking water and has the highest *per capita* consumption of black tea in the world. Tea is a hyperaccumulator of fluoride and chronic fluoride intake is associated with multiple negative health outcomes. In this study, fifty four brands of the commercially available black tea bag products were purchased and the fluoride level in tea infusions tested by an ion-selective electrode method. The fluoride content in all brands tested ranged from 1.6 to 6.1 mg/L, with a mean value of 3.3 mg/L. According to our risk assessment it is evident that the general population in the RoI is at a high risk of chronic fluoride exposure and associated adverse health effects based on established reference values. We conclude that the culture of habitual tea drinking in the RoI indicates that the total cumulative dietary fluoride intake in the general population could readily exceed the levels known to cause chronic fluoride intoxication. Evidence suggests that excessive fluoride intake may be contributing to a wide range of adverse health effects. Therefore from a public health perspective, it would seem prudent and sensible that risk reduction measures be implemented to reduce the total body burden of fluoride in the population.

## 1. Introduction

Fluoride is a halogen found in soil, water, rocks, air, plants and animals in different quantities [1]. The major sources of internal exposure of individuals to fluorides are the diet, dental products, dermal absorption from chemicals or pharmaceuticals, ingestion or parenteral administration of fluoride-containing drugs, tobacco consumption, exposure to industrial emissions and ingestion of fluoride-containing soil [2]. It is now acknowledged that of all the common foodstuffs, tea has one of the highest potentials for increasing the daily fluoride intake [2,3,4]. Indeed, the tea plant, *Camellia sinensis* has been known since the 1930s to be a hyperaccumulator of fluoride [5,6]. Tea trees accumulate and store fluoride by absorbing it from the air and soil. Fluoride accumulates mostly in the leaves of the tea plant [2]. A substantial amount of fluoride is released during tea infusion. Because soluble fluoride is easily absorbed by the gastrointestional track, the bioavailability of fluoride from tea is close to 100% [7] which is similar to that from drinking water [8]. Only the water-soluble ionic fluoride ingested is relevant to human health.

Fluoride has no known essential function in human growth and development and no signs of fluoride deficiency have been identified in humans [9]. The role of fluoride in the prevention of caries has been known for many years. Though fluoride is not essential for tooth development, fluoride can contribute to the prevention of caries [10,11], but caries is not a fluoride deficiency disease [9]. The benefits of fluoride for the prevention of dental caries has lead to the addition of fluoride into toothpaste, water, milk and salt so as to provide its desired amount to the population. However, scientific evidence suggests that the widespread use of topical fluoride sources, such as toothpastes, is the most effective in preventing dental caries [12,13]. Notwithstanding this, the wider health effects of fluoridation remain controversial. Fluoride has a very narrow safety margin between its optimum benefit for caries prevention and chronic fluoride intoxication. As fluoride is available from various sources for human consumption, the total ingestion of fluoride by a person should be estimated taking into consideration the fluoride consumed from all the sources, to get either the beneficial effect or to prevent the deleterious effect [14]. The major natural source of fluoride emissions are atmospheric volcanic emissions and volcanic aquifers. In certain geographic regions exposure to fluoride from volcanic activity and groundwater can be significant resulting in chronic fluoride intoxication. Since the 1950s, in a few developed countries, exposure to fluoride has changed significantly with the introduction of artificial water fluoridation. This has resulted in increased dietary intake from water, from foods cooked with fluoridated water as well as from consumption of processed foods and beverages reconstituted with fluoridated water. Since the 1980s, salt fluoridation programmes have also been introduced in a number of countries particularly in Central and South America, as well as in some European countries. At the same time, the use of fluoride in dental products such as fluoride toothpaste, gels, varnishes, glass ionomer cements and sealants and the dramatic increase in the use of fluorine in medical pharmaceuticals has contributed to further exposure of the population. In addition to dietary sources of fluoride, rapid industrialisation and changes in agricultural practices has resulted in increased anthropogenic emissions of fluoride. Over the last century, however, the one relatively constant source of dietary exposure has been tea. In this paper, we discuss excessive exposure of fluoride from tea, the most widely consumed beverage in the Republic of Ireland (RoI).

Fluoride is a cumulative toxin [15]. Along with urinary excretion, uptake by bone removes excess fluoride from circulation, thus effectively removing fluoride from the fluids bathing the soft tissues [16]. In infants retention in bone can be as high as 90% of the absorbed amount, whereas in adults bone retention is 50% or less [17]. Bone fluoride concentrations increase with both magnitude and length of exposure [2]. The clinical course of fluoride intoxication (fluorosis) involves a number of factors including kidney function, age, sex, calcium intake, dose and duration of fluoride intake [15]. Fluorosis involves dental, skeletal and non-skeletal effects [18,19]. Increased fluoride bone content is thought to be the main indicator of chronic fluoride poisoning [20]. Mostafaei *et al.* found that the rate of increase in fluoride bone levels is almost 3-fold higher among tea drinkers compared to non-tea drinkers [21]. Non skeletal effects of fluoride include cardiotoxicity [1,22,23,24,25,26,27,28,29], neurotoxicity [2,30,31,32,33,34,35,36,37], endocrine dysfunction [1,2,38,39,40], hepatotoxicity and nephrotoxicity [41,42].

In recent years a number of other studies have highlighted the safety concerns regarding the exposure to a high levels of fluorides in black teas [43,44,45,46,47,48,49,50,51]. Tea drinking in infancy is known to cause dental fluorosis [52,53,54,55] as well as early stage skeletal fluorosis [56]. Different authors have drawn attention to skeletal fluorosis among adults induced by habitual tea drinking in certain regions such as the USA, China, Tibet, Mongolia, France and the UK [52,57,58,59,60,61,62,63,64,65,66,67,68,69,70,71,72,73] and that induced, on the other hand, by prolonged ingestion of mineral water with high fluoride concentrations [74,75,76,77,78,79,80,81], the use of certain fluoridated medications [82,83,84,85,86], by the ingestion of fluoride toothpaste [87] and from administration of sodium fluoride in the treatment of osteoporosis [88,89,90,91,92].

However, while authorities in the US and the European Union have imposed restrictions on the fluoride content in bottled mineral waters [93,94], no action has been made to minimize the fluoride content in tea. It is now clear that black tea bags, as well as other types of tea products, have particularly high levels of fluoride, which can pose a significant health risk if tea is consumed in significant quantities [45]. Research has indicated that among populations habitually consuming black tea, water fluoridation is not only unnecessary but also possibly harmful [44].

The RoI has the highest *per capita* consumption of tea in the world [95,96]. In Ireland, habitual tea drinking reached “prodigious levels” by the 20th century [97] and tea remains the most frequently consumed beverage, consumed by 91% of adults aged 18 to 64 years of age [98]. The 2010 National Longitudinal Study of Children in Ireland reported that 8% of infants were given tea to drink at nine months of age. Among the lower socioeconomic class the percentage was 10% [99]. An earlier study by Freeman (1996) reported that at 18 months of age 12% of infants were drinking tea from a bottle [100]. 

In the RoI people predominantly drink black tea with 95% of the tea being sold in tea bags. The loose tea leaf market is very small, about 3% to 4% [101]. In 1990, *per capita* tea consumption was 3.21 kg per person [102] and 2.96 kg in 2003 [103]. Overall, *per capita* tea consumption is 6-fold higher than the global average [101].

Differences in the fluoride content of tea leaves can be attributed to the geological, soil chemical and physical characteristics of the area of production [104]. The water extractable fluoride content depends on a number of factors including the fluoride content of the water used to prepare the beverage, the brand of tea, leaf size, infusion time and temperature of infusion. [103,104,105,106]. Drinking water supplies in the RoI are artificially fluoridated since the 1960s (0.8–1.0 mg/L, reduced to 0.6–0.8 mg/L in 2007), thus, the fluoride content in tea infusions in the RoI also contains fluoride from drinking water. Since the 1960’s most Irish tea products are primarily sourced from East Africa, accounting for 80% of the total tea imports. East of the Rift valley in Kenya is the largest source of tea [107]. Recently, authorities in China imposed restrictions on most of Kenya’s tea products due to potentially hazardous concentrations of fluoride found in some of them [108].

Although the Irish are the heaviest tea drinking nation in the world, the fluoride levels in black tea commodities consumed by the population has never been evaluated [109]. The European Food Safety Authority (EFSA) reported that drinking just 2 cups of tea per day (with a fluoride content of 5 mg/L), combined with an average consumption of fluoridated drinking water and the use of fluoridated tap water in the preparation of food, but excluding all other sources (including solid foods, toothpaste and dental products), would provide a daily dietary intake of 6 mg fluoride per day [9]. In the EU, the Tolerable Upper Intake Level (UL) for fluoride has been established at 1.5 mg/day for children aged 1–3 years, 2.5 mg/day for children up to the age of eight years, 5 mg/day for children aged 9 to 14 years and 7 mg/day for children older than 15 years and adults [110]. Considering that the average person in the RoI consumes 4–6 cups of tea per day and that drinking water is artificially fluoridated to a concentration ranging from 0.6 to 0.8 mg/L (reduced from 1.0 mg/L in 2007), this would strongly suggest that the majority of the population in Ireland are at risk of chronic fluoride intoxication. As no information is available on the fluoride content in black tea marketed and consumed in the RoI an exposure and risk assessment is required. The objectives of this study were to determine: (1) infusible fluoride levels of marketed black tea bag products consumed by the population; (2) the contribution of tea consumption to Adequate Dietary Intake and Tolerable Upper Intake levels; and (3) the reasonable maximum exposure to fluoride from tea consumption and associated health risks. The study does not include fluoride intake from other beverages, foods, dental products or medications.

## 2. Methods

### 2.1. Sampling

In total 54 of the most widely consumed brands of black tea products were procured as boxes of tea bags from supermarkets, retail outlets and health food stores in the RoI. The products included all the leading consumer black tea bag products as well as supermarket store-brand tea products. All samples were tea bag products sold in boxes which represent the vast majority of tea products purchased by consumers. The range of tea samples tested included caffeinated, non-caffeinated and organic black tea products. We did not consider other types, such are green, oolong, white, yellow or herbal tea, which are not widely consumed. For each product the details of the origin of the tea as marketed was noted and the weight of tea bags determined. The characteristics of the analyzed teas are listed in Table 1.

### 2.2. Preparation of Tea Infusions

In order to determine the fluoride concentrations in tea infusions, two tea bags were selected at random from each box and samples prepared using freshly boiled fluoridated drinking water, with a fluoride level of 0.7 mg/L. Public water supplies were used so that fluoride levels would be representative of ingested tea as prepared in the home, in workplaces or food service settings. Each tea bag was brewed for a period of 5 min with 200 mL of water initially at 100 °C. After five minutes, the tea bag was taken out and the infusion allowed to cool to room temperature. To remove excess water tea bags were squeezed lightly with a spoon before removal, as is generally the method used by consumers when preparing a cup of tea with disposable tea bags. When the solution reached room temperature a 20 mL aliquot was taken from each infusion and placed in a polyethylene plastic sample container.

### 2.3. Fluoride Analysis

For every sample, a Total Ionic Strength Adjustment Buffer (TISAB) tablet (FL704 TISAB Tablets Extech, Nashua, NH 03063, USA) was added to each 20 mL aliquot. The fluoride concentrations in the so-prepared samples were determined in duplicate with a fluoride ion selective electrode (Extech FL700). The Extech FL700 has a reported resolution of 0.1 mg/L and overall accuracy of ±3%. A series of fluoride standards were prepared by using a 100 mg/L fluoride Eutech standard solution (Fluoride Standard Solution Code No. ECSCSFL3BT, Eutech Instruments, supplied by Lennox Laboratory Supplies, Ireland) by diluting appropriate volumes to 100 mL with de-ionized water. Then, the electrode was calibrated to concentrations of 0.0, 0.5, 1.0, 5.0, and 10.0 mg/L. Prior to sampling and at the end of group of five samples, the electrode was re-checked for accuracy.

### 2.4. Quality Assurance

The entire analytical procedure for the determination of the ionic fluoride concentration in tea infusions was first tested using public water supplies with a fluoride level of 0.7 mg/L. For quality assurance tea infusions were further prepared using boiled deionized water following the same methodology. In total, 162 individual infusions of tea were prepared. The results show that the difference was 0.70 mg/L. The results of these analyses are presented in Table 1.

## 3. Results

Table 1 provides the list of tea products tested and the county of origin as listed on product labels. The weight of tea bags and fluoride levels in tea infusions after 5 min brewing are also listed in Table 1. For the duplicate tea infusion samples the mean value is provided. The variability between duplicate samples was very small, generally within 0.1 mg/L.

When tea infusions were prepared with tap water the fluoride concentration in tea infusions ranged from 1.6 to 6.1 mg/L with a mean of 3.3 mg/L. All tea products tested exceeded 1.5 mg/L. 96% of the products exceeded 2.0 mg/L, 59% exceeded 3.0 mg/L, 24% exceeded 4.0 mg/L and 7% exceeded 5.0 mg/L. As expected, the use of fluoridated tap water was found to increase the measured fluoride content in all tea infusions. When tea was prepared with deionized water 22% of tea products had a fluoride level exceeding 3.0 mg/L compared to 59% when prepared with tap water. Notwithstanding the contribution of fluoridated water, even when prepared with deionized water, 96% of the tea products had fluoride concentrations that exceeded 1.5 mg/L. Overall, the lowest fluoride levels were found in pure tea products sourced from Assam, India. These products are lesser known brands not widely available or consumed in the RoI. The fluoride level in tea infusions from six decaffeinated black tea products tested ranged from 1.6 to 4.2 mg/L when prepared with tap water and 0.8 to 3.2 mg/L when prepared with deionized water. The lowest fluoride level was found in organic decaffeinated tea from Assam, India. 

Of the four caffeinated organic tea brands tested, the fluoride level was found to range from 2.0 mg/L to 4.2 mg/L when prepared with tap water. The highest fluoride level was present in an Irish blended brand, originating from Kenya; the lowest level was found in organic Clipper Assam tea from India. The study identified differences in fluoride levels across the same brand, with different blended teas containing variable levels of fluoride. It is expected that the variety of tea used for blending, the percentage composition of blended tea, the particle size of tea and the different country of origin of tea leaf used for blending would alter the fluoride content in blended teas. The study also identified a lack of reporting of the country of origin of tea, no data was provided on fluoride levels of tea products and no guidelines or other recommendations were provided regarding the safety of drinking tea for infants, pregnant mothers or the general public. The results of this study also identified that large regional variations exist in the packaging and portion size of tea sold internationally. For example, in this study, it was found that the average weight of a single tea bag from tea products in the RoI was 3.3 g. In the U.S. the industry standard weight for individual cup-size teabags is just 2.2 g [111]. This difference reflects consumer preference for strong tea in the RoI.

### 3.1. Exposure Assessment

There is some controversy regarding recommendations on AI for fluoride since there are no signs of fluoride deficiency which have been identified in humans, and fluoride has no known essential function in human growth and development [9]. Evidence over the last 20 years has demonstrated that the cariostatic effect of fluoride is topical on the tooth surface and ingestion is not required [12,112,113]. In 2011, the EFSA commissioned the University of East Anglia to examine the scientific data from which Dietary Reference Values (DRVs) for fluoride may be derived. The comprehensive review found that there were relatively few studies of good quality regarding fluoride intake, accumulation and/or health endpoints. The review concluded that there was a lack of high quality evidence upon which DRVs may potentially be based for fluoride [114]. Notwithstanding this, the EFSA have recommended AI’s for fluoride [9,16], which are similar to those suggested by the U.S. Institute of Medicine [115]. Table 2 provides details of dietary reference values.

According to the EFSA an AI is the average observed daily level of intake by a population group (or groups) of apparently healthy people that is assumed to be adequate, while chronic intakes above the Tolerable Upper Intake Level (UL) may be associated with an increased risk of adverse effects [116]. In general, the AI for fluoride are based on estimated intakes that have been shown to reduce the occurrence of dental caries maximally in a population without causing unwanted side effects including moderate dental fluorosis [117]. The AI and UL applies to intake from all sources including water, beverages, foodstuffs, dental health products and drugs. SCHER reported that the emerging picture from all risk assessments conducted on fluoride is that there exists a narrow margin between the recommended intakes for the prevention of dental caries and the upper limits of exposure [13]. In view of the different health, nutritional status and habits of individuals in addition to the differing consumption of drinking water and the uncontrolled intake of fluorides from other sources, such as tea, medications or occupational exposures, it is not possible to accurately define an AI or UL for a population without excluding sensitive subgroups of the population. To illustrate this dilemma, the intake of fluoride for individuals with low iodine status or poor renal function poses significantly higher risk of negative outcomes than for healthy individuals [2]. Furthermore, in countries with artificial fluoridation, a large proportion of very young children who are bottle fed with powdered infant formula reconstituted with tap water may already be at risk of excess fluoride intake without taking other dietary sources of fluoride into account. The EFSA acknowledged that the UL would be exceeded in infants if water containing more than 0.7 mg/L is used for preparation of the formula [17]. It is important to consider that breast feeding prevalence in Ireland is among the lowest in the world and the majority of infants may potentially already exceed the UL through infant formula alone. In our study, fluoride exposure through consumption of tea was calculated using a deterministic approach. The dose of fluoride in 150 mL, 250 mL and 750 mL, 1 L, 1.5 and 3.8 L of tea infusions for each brand of tea was calculated based on the measured fluoride content (mg/L). The total fluoride intake by volume of tea consumed was compared to the daily AI and UL intake reference values. The mean of all 54 brands tested is provided along with range of fluoride intake for all brands. Based on a range of fluid intakes the percentage contribution of each brand of tea to the AI and UL is presented for infants, children and adults.

### 3.2. Infants and Children

The exposure assessment presented here is specific to tea as prepared with tap water and excludes all other sources of dietary fluoride intake. In our assessment of fluoride exposure from tea a number of scenarios were examined. While it has been reported that up to 10% of infants in the RoI drink tea at nine months of age there are no data to estimate tea intakes at single doses or daily intakes for children for the RoI. In Israel, the consumption of tea among infants aged 6–12 months were reported to vary between 50 and 750 mL (mean 250 mL per day) [118]. In the Australian 2008 total diet study, tea consumption was also reported among infants and children; mean daily intake was approximately 150 mL for infants aged 1–3 years; 250 mL for children aged 4–8 years; 300 mL for 9–13 year olds and 500 mL for 14–18 year olds [119]. The EFSA reported that tea consumption was a major source of caffeine intake in toddlers and children up to ten years of age in a number of European countries including Bulgaria, Italy, Denmark, Netherlands and the UK. However, no data was provided for the RoI [120]. Considering that tea consumption in the RoI significantly exceeds all other European countries, as well as Israel and Australia, it is expected that individual consumption by infants and children could be higher in the RoI. In this study the fluoride intake from consumption of 150 mL, 250 mL and 750 mL were evaluated.

Based on our data, we can assume that there is a significant risk for an excessive intake of fluoride from tea consumption could contribute intakes above the recommended AI and exceed the UL. Our data shows that 150 mL of tea can exceed the AI for an infant aged 7–11 months 2 fold and up to 1.5 fold for children aged 1–3 years. In the EU no UL has been established for infants under 1 year of age, however the IOM have established a UL for infants aged 0–6 months of 0.7 mg per day and 0.9 mg per day for infants aged 4–12 months [115]. Based on IOM recommendations just 150 mL of tea could provide up to 1.3 fold of the UL for an infant aged 0–6 months and 1.1 fold of the UL for infants aged 4–12 months. Similarly, based on IOM recommendations one cup or infant bottle of tea could exceed the UL for infants aged 0–6 months and 4–12 months by 2.2 fold and 1.7 fold respectively. A summary of the exposure and risk assessment intakes are presented in Table 3.

### 3.3. Adults

As with infants and children, the exposure assessment is specific to tea as prepared with tap water and excludes all other sources of dietary fluoride intake. The value for total consumption of water-based beverages across European countries ranges from about 700 mL/day/person at the lowest reported mean to about 3800 mL/day/person at the highest reported 97.5th percentile [13]. In the RoI, tea is consumed by more than 90% of the adult population, with the average person consuming 4–6 cups per day. In this study, four scenarios are examined, consumption of 250 mL (about 1 cup) per day, 1 litre per day (4 cups or 3 mugs of tea per day), 1.5 L per day (6 cups or 4 mugs per day) and a reasonable maximum intake of 3.8 L per day (15 cups or 10 mugs per day). The Reasonable Maximal Exposure (RME) is considered conservative as a number of studies have reported individuals consuming 3.5–7.5 L of black tea per day [60,61,62,63,121,122]. A summary of exposure and risk estimates for fluoride intake are presented in Table 4. The range of exposures for all products tested and the mean values are provided for each scenario.

The findings of this study demonstrate that 250 mL or roughly one cup of tea when prepared with tap water can provide between 0.4 and 1.5 mg fluoride to daily dietary intake. Of the 54 tea products tested 15 teas would provide 1 mg/L fluoride or more per 250 mL serving. One cup of tea can provide up to half of the AI for an adult female and 44% for an adult male. Based on the UL of 7 mg per day as recommended by the EFSA one cup of tea can provide up to one fifth of the UL.

Assuming a daily intake of 1 L (4 cups of tea) per day, 32 of the 54 tea products tested (59%) would exceed the AI for a healthy adult female and 19 (35%) would exceed the AI for a healthy adult male. The tea product with the highest fluoride concentration would provide more than 2-fold the AI for a female and 1.8-fold the AI for a male, respectively. In consideration of the tolerable upper intake level (UL) as recommended by the EFSA, the consumption of four cups of tea could provide up to 86% of the UL for a healthy adult (either sex) and could readily exceed the UL for children aged 9–14 years. Assuming a daily intake of six cups per day all but one of the teas (98%) would exceed the AI for an adult female and 48 out of 54 (88%) would exceed the AI for a healthy adult male. The tea product with the highest fluoride concentration would exceed the AI for a female 3-fold and 2.6-fold for an adult male. Consuming six cups of tea would exceed the UL for an adult 1.3-fold and up to 1.8- fold for children aged 9–14 years, respectively. Almost 10% of the teas would exceed the UL established for all dietary fluoride sources. In considering the RME from tea, the results of this study demonstrate that the mean RME for an adult living in a fluoridated community, based on the average of all brands tested, would be 12.4 mg/day, or 70% above the recommended UL. If a person used the black tea product with the highest fluoride content the maximum RME from tea consumption would be as high as 23 mg per day. This is similar to that reported by Whyte and associates [61].

## 4. Discussion

The purpose of the present study was to determine the fluoride concentrations in black tea infusions in the RoI, the potential dietary exposure to fluoride from tea and associated health risks. Our results demonstrate that the high fluoride levels measured in black tea prepared from packaged black tea bag products sold in the RoI are consistent with concentrations found in similar products sold in Taiwan [43,46]; China [44,45]; Poland [47]; Slovenia [49]; UK [51]; Germany [123]; Norway [124] and the USA [125].

In the RoI, the legally enforceable upper limit for fluoride in artificially fluoridated water is 0.8 mg/L and 1.5 mg/L for potable water with naturally occurring fluoride. Within the EU, the enforceable upper limit for all drinking waters is 1.5 mg/L [126]. As shown in Table 1, all of the black teas had fluoride concentrations exceeding the enforceable level in drinking water. In addition, all products exceeded the maximum permitted level requiring labelling and safety precautions for bottled mineral water under European regulations [94]. Moreover, four products (7.4%) exceeded the fluoride concentration which the EU has found to pose a risk to public health if present in bottled mineral water [94]. Based on the fluoride levels in tea and reference levels for AI and UL our results suggest that the general population in the RoI is at risk of excessive intake of fluoride and that fluoridation of drinking water further contributes to the risk of fluoride intoxication. The implications of the findings of our study are clear:

(1) Tea should not be consumed by infants or children, to prevent the risk of cardiotoxicity, neurotoxicity, dental fluorosis, abnormal bone metabolism, endocrine dysfunction, hepatotoxicity and nephrotoxicity. It is a public health concern that all tea products tested exceeded the fluoride levels reported in the literature to be associated with a lowering of the IQ in children [30,37] when prepared with tap water and found to be within the range of values reported to cause neurotoxicity [31,32,33,34]. Our data also indicates that tea consumption by infants will result in excessive intake of fluoride which is associated with a high risk of dental fluorosis. Recently, Chen *et al.* provided a biological dose-exposure response relationship for fluoride exposure and bone metabolism indicators and demonstrated that when urinary fluoride levels increased above 0.3 mg/L, the percentage of children with abnormal bone metabolism increased significantly [127]. Székely *et al.* examined urinary fluoride excretion after tea consumption in young adults residing in a community with a fluoride level in drinking water of 0.1 mg/L and determined that the consumption of 200 mL of tea per day with a fluoride content of 0.42 mg increased 24 h urinary fluoride levels from 0.297 mg/L to 0.451 mg/L [128]. Urinary fluoride levels in children and adolescents in communities with water fluoridation are significantly higher than those reported in the latter study. For example, in North America where tea drinking is relatively uncommon, urinary fluoride levels generally exceed 1 mg/L [129]. Clearly the consumption of tea by children in communities with water fluoridation would contribute to even higher urinary fluoride concentrations. Moreover, the concentrations would exceed the levels reported in literature to be associated with cardiotoxicity and endocrine disruption in children [1,37]. Based on the fluoride levels present in tea in our study, it is also evident that consumption of tea can contribute to abnormal bone metabolism in children. Furthermore, 96% the tea products exceeded the level that has been reported in literature to cause liver and kidney damage in children and 24% exceeding this level 2-fold [41,42].

(2) Considering fetal effects, fluoride can cross the placenta into the fetus and high intake of fluoride during pregnancy can be harmful to the developing child. Du *et al.* demonstrated the passage of fluoride through the placenta of mothers with high fluoride intake and its accumulation within the brain of the foetus has impacts on the developing central nervous system and brain [130]. Strunecka *et al.* reported that fetal exposure to fluoride adversely affects brain development and that chronic fluoride exposure in the course of intrauterine fetal life may produce certain harmful effects on the developing brain of the foetus [131]. High fluoride intake from tea consumption in pregnancy may also impair bone health in offspring. Shi *et al.* demonstrated that maternal fluoride intake presented a dose/response relationship to fluoride levels as seen in bone fluoride levels in foetuses. When the latter reached greater than 500 μg/g, pathological changes occurred in 90% of the bone [132].

(3) As fluoride accumulates in bone progressively over many years, it is expected that the total body burden of fluoride in the general population in the RoI will be very high by international standards. The results of our study indicate a significant percentage of the general population are at risk of chronic fluoride intoxication. It is known that when excessive amounts of fluoride are consumed, it tends to accumulate more in cancellous bone compared to cortical bone [133]. As the cancellous bone predominates in the region of joints, the higher fluoride accumulation leads to damage to the bone matrix with resultant complications [134]. The NRC reported that in the absence of other sources of fluoride, the consumption of drinking water with a fluoride level of 1 ppm, bone fluoride levels can reach 2500 mg/kg in twelve years and to 3000 to 4000 mg/kg over longer periods [2]. Symptoms of clinical stages I and II skeletal fluorosis may occur with bone concentrations above 3500 ppm [2]. A lifetime exposure to fluoride at 2 mg/L would fall within or exceed the ranges of concentrations that have been associated with stage II and stage III skeletal fluorosis [2]. Stage II skeletal fluorosis is associated with chronic joint pain, arthritic symptoms, slight calcification of ligaments, and osteosclerosis of cancellous bones. Stage III has been termed “crippling” skeletal fluorosis because mobility is significantly affected as a result of excessive calcifications in joints, ligaments, and vertebral bodies [2]. As noted previously, in a recent Canadian study Mostafaei *et al.* demonstrated the rate of increase in fluoride bone levels is almost 3 fold higher among tea drinkers compared to non-tea drinkers [21]. Of note, Weatherell measured the fluoride levels in *femoral compacta* from humans of different ages who lived in communities supplied with drinking water containing less than 0.5 ppm fluoride in various locations in England and Rochester, New York. Significant differences were found between the two populations, with fluoride accumulating to 4000 ppm among the English subjects compared to less than 1000 ppm among residents in Rochester [135]. Since drinking water in both communities was unfluoridated (<0.5 ppm) the fourfold difference in bone fluoride levels reported may largely reflect the higher consumption of tea in England. Considering that drinking water has been artificially fluoridated in the RoI for over 50 years and tea consumption is 20% higher than the UK and sevenfold higher than Canada, it is evident that the population in the RoI may be at a higher risk of chronic fluoride intoxication, which would present primarily as musculoskeletal disorders, arthritic-like symptoms with chronic joint pain and disability in later life. Musculoskeletal conditions are indeed highly prevalent in the RoI and their impact is pervasive. The burden of musculoskeletal disorders in the RoI can be measured in terms of the economic cost of chronic pain to the RoI, which was reported to be €5.34 billion in 2012 [136].It is reported that over 900,000 people, or 1 in 5 persons, in the RoI are affected by arthritis [137]. Research has found a significant association between daily fluoride intake and lower back pain (LBP) [138]. The authors suggested that this could be deemed as the early stage of mild skeletal fluorosis caused by joint or bone degeneration because of chronic fluoride exposure. In 2010, it was estimated that 395,000 adults in the RoI (11.9%; 95% CI = (8.8%, 14.9%)) had lower back pain or another chronic back condition in the previous 12 months that had been clinically diagnosed. The Institute of Public Health reported that this is likely to be an underestimate of the total number of people with these conditions [139].

(4) The findings of this study also show that for the general population who consume tea daily, fluoride exposure can significantly exceed the levels reported by the NRC that can cause thyroid dysfunction in persons with either low or adequate iodine intake [2]. For individuals with insufficient iodine intake, the NRC reported that thyroid function may be impaired at a total fluoride intake of between 0.7 and 2.1 mg per day for a 70 kg individual, or alternatively, at fluoride intakes of 3.5–9.1 mg per day, when iodine intake is adequate [2]. This would suggest that regular consumption of modest amounts of tea by itself may deliver sufficient fluoride to contribute to thyroid dysfunction. Indeed, this phenomenon was documented in a recent study conducted in Saudi Arabia [38,39]. Importantly, tea was identified as the main dietary source of fluoride contributing up to 3 mg/day among adults, 1.27 mg/day among some adolescents and 1.02 mg/day among some children. [39,40]. Our data also suggests that tea is also the major source of fluoride intake in the population in the RoI. However, per capita tea consumption in the RoI is 5-fold higher than in Saudi Arabia, in addition drinking water supplies are fluoridated in the RoI and non-fluoridated in Saudi Arabia. This suggests that endocrine disorders may be even more prevalent in the RoI. The possibility of thyroid dysfunction is also increased in Ireland because it is a country at risk for iodine-deficiency [140]. It is generally accepted that iodine deficiency may enhance the toxicity of fluoride [2]. Iodine deficiency has been found to be highly prevalent in Ireland [141]. The specific incidence of thyroid disorders is uncertain in the RoI, however, the National Centre for Pharmacoeconomics reported that the third most prescribed drug under the Government Medical Services scheme in the RoI is levothyroxine, used for the treatment of hypothyroidism [142]. This data suggests that thyroid disorders are decidedly widespread among the population. Interestingly, one recent UK study found significantly higher rates of hypothyroidism in communities with artificial fluoridation compared to non-fluoridated communities Although tea consumption was not included as a confounding factor in this study; it is likely that tea consumption patterns would be evenly proportioned among the study populations. Notwithstanding this, the community with artificial fluoridation would be expected to have higher dietary fluoride intake.

(5) Several studies have reported that fluoride can cause cardiovascular system dysfunctions in humans mainly presenting as arrhythmias [1,22,23,24,25,26,27,28,29]. It is known that cardiac arrhythmias contribute significantly to ischemic heart disease and risk of sudden cardiac death [143,144]. These findings are clearly relevant to the RoI not only because of the high fluoride intake of the population, but because mortality rates from ischemic heart disease have been found to be significantly higher in the RoI than the European average [145].

(6) Several subpopulations are likely to be more susceptible to the toxic effects of excessive fluoride intake. It is known that the effect of fluoride is more marked in populations with malnutrition who have low calcium, vitamin D, and protein intakes [146]. Malnutrition is a common clinical and public health problem in the RoI [147]. Rice *et al.* reported that the annual public health and social care cost associated with adult malnourished patients in Ireland is estimated at over €1.4 billion, representing 10% of the health-care budget [148]. Vitamin D and calcium deficiency is also highly prevalent in the RoI across all age groups. The Irish Universities Nutrition Alliance (IUNA) reported that among people aged 18–64 years of age there was a significant prevalence of inadequate calcium intake and a substantial proportion of the population had inadequate Vitamin D intakes [98]. Recently, the National Medicines Information Centre reported that it has been estimated that in Ireland 74% of adults have <50% of the recommended daily intake of Vitamin D [149]. Another subgroup of the population at risk of fluoride toxicity are individuals with reduced renal function. Among adults and children with impaired renal function, the ability to excrete fluoride markedly declines, resulting in greater retention of fluoride in the body and higher plasma and bone fluoride concentrations. It is known that the onset of the appearance of clinical fluorosis is much shorter in patients with renal impairment [77,80,81,150]. In 2003, in the RoI an estimated 200,000 people had diabetes and a further 200,000 had the condition but were unaware of it. This corresponds to 9.6% of the general population having diabetes. Moreover, it was also estimated that a further 250,000 had pre-diabetes and 50% would develop diabetes in the ensuing 5 years if lifestyle changes were not met [151].

(7) In the EU, Regulation (EC) No 178/2002 provides the general principles and requirements of food law (General Food Law Regulation) [152]. The General food law is applicable to all foodstuffs, including tea. In determining whether any food is unsafe, the regulations specify that regard shall be had to the normal conditions of use of the food by the consumer and to the information provided to the consumer, including information on the label, or other information generally available to the consumer concerning the avoidance of specific adverse health effects from a particular food or category of foods [152]. In determining whether any food is injurious to health, the regulations specify that regard shall be given to the probable immediate and/or short-term and/or long-term effects of that food on the health of a person consuming it; to the probable cumulative toxic effects and to the particular health sensitivities of a specific category of consumers where the food is intended for that category of consumers. The regulations also specify that food law shall aim at the protection of the interests of consumers and shall provide a basis for consumers to make informed choices in relation to the foods they consume [152]. Based on current evidence, the tea products tested in this study do not comply with EU food law.

(8) Decisions about water fluoridation should include the total amount of fluoride intake from all sources including foods, beverages and medications such that cumulative effects and the risk of chronic fluoride intoxication are reduced. National government decisions on risk management should have as their primary objective the protection of the health of consumers [153]. In the EU, the precautionary principle is a fundamental part of risk management [154]. Paradoxically, while fluoridation of drinking water is regarded as the controlled addition of fluoride to drinking water, requiring constant surveillance and monitoring, it is apparent that there has been little or no surveillance in the RoI of total dietary fluoride intake, total body burdens of fluoride or residual levels of fluoride in food. Thus, the effect of fluoridation of drinking water in the RoI is that it has resulted in compounding safety factors associated with uncontrolled exposure and contributed to excessive accumulation of fluoride in the general population.

(9) The most obvious and the earliest clinical manifestation of fluoride toxicity is dental fluorosis, but it only records the effects of ingestion in the first six years of life [155]. A systematic review carried out using data from nine countries (Australia, Canada, Finland, Ireland, Italy, New Zealand, Sweden, Britain, and the USA) by York University (UK) concluded that dental fluorosis would likely occur in 48% (95% CI: 40%–57%) of the population exposed to municipal water fluoridated at the level of 1 mg/L [156]. Cochran *et al.* in a comparative study of the prevalence of dental fluorosis in 8-year-old children from seven European study sites using a standardized methodology, reported the prevalence of any fluorosis at 89% among children in the RoI [157]. Additional risks of increased fluoride exposure are known, including skeletal and non-skeletal fluorosis. It is now recognized that excess fluoride intake can contribute to musculoskeletal disease [2,52,56,57,58,59,60,61,62,63,64,65,66,67,68,69,70,71,72,73,74,75,76,77,78,79,80,81,82,83,84,85,86,87,88,89,90,91,92,133,134] neurotoxicity [2,30,31,32,33,34,35,36,37], cardiotoxicity [1,22,23,24,25,26,27,28,29], endocrine dysfunction [1,2,38,39,40], hepatotoxicity and nephrotoxicity [40,41]. The EU AI and UL values for children up to eight years of age are based on preventing dental fluorosis, while the UL value in older children and adults is based on data from observational and intervention studies with regard to bone fractures [9]. However, research has indicated that moderate fluoride intake is associated with endocrine dysfunction [39,40]. Further research has demonstrated that fluoride induced hypothyroidism is associated with cardiotoxicity in children [1]. Moreover, a current study by Zhang *et al.* demonstrated that when urinary fluoride levels exceeded 0.5 mg/L and serum fluoride levels exceeded 2.6 µM, declines in children’s IQ can occur [37]. Based on current evidence there is an increasing need to develop revised guidelines on the nature and severity of risks associated with excessive fluoride intake in order to better protect human health. While our study is the first to quantitatively measure the fluoride content and estimate fluoride exposure from tea in the RoI, it nevertheless only uses a single risk factor (tea consumption) as a basis for fluoride exposure. Given, as we have indicated, that the fluoride from tea consumption has already potentially overburdened the RoI population any additional sources would compound the effect. The body burden of fluoride is best reflected in bone fluoride concentrations which provide the best indicator of long term fluoride exposure and body burden [155]. No data, however, is available on biomonitoring for fluoride exposure or age related fluoride accumulation among the adult population in the RoI and no studies have been conducted to examine the association between fluoride intake or accumulation and disease prevalence. Available evidence, however, strongly suggests that there is a high burden of dental fluorosis, musculoskeletal disorders, thyroid disorders, and ischemic heart disease in the RoI. Furthermore, evidence suggests that there is a large subgroup of the population particularly susceptible to the adverse effects of chronic fluoride intake due to low iodine intake, malnutrition, Vitamin D deficiency, calcium deficiency and diabetes prevalence.

### 4.1. Recommendations

With regard to the findings in this study, the authors have the following recommendations. To begin, health care and regulatory officials need to be more aware of dietary sources of fluoride and the clinical symptoms of fluoride intoxication. All patients presenting to clinicians with musculoskeletal or endocrine disorders should also be assessed as to their total dietary fluoride intake. To reduce the risk of chronic fluoride toxicity, public health authorities should inform pregnant women that habitual tea drinking is unsafe for the developing child and that tea should not be provided to infants as a beverage. Given the very high fluoride levels found in many tea products there is a need for the establishment of maximum levels allowed in tea. The findings of our study highlight the difficulties in managing exposures from multiple sources of fluoride. Appropriate actions should be taken by public health authorities to reduce direct exposure as well as body burden of fluoride in the general population. The most immediate action would be to cease fluoridation of drinking water supplies.

### 4.2. Limitations of the Study

The objective of this study was to determine the fluoride content in tea infusions using an established and recognized methodology. The most widely used method of fluoride quantification has involved potentiometry employing the fluoride ion selective electrode (ISE) [158]. It is noted that our results are similar to those reported in published literature [42,43,44,45,46,47,48,49,50,51,52,53,54,55,56,57,58,59,60,61,62,63,64,65,66,67,68]. However, the possible interference of aluminium (Al) with fluoride measurement using ISE procedures was not examined in any of these studies. Our review of literature found that high Al concentrations exceeding 2.5 mg/L have been observed to result in under-representation of fluoride levels in tea infusions when measured by ISE method [159,160]. During the course of our study the Al content was measured using Inductively Coupled Plasma Atomic Emission Spectroscopy (ICP-AES) in a subsample of six tea infusions and one sample of drinking water by a third party laboratory. All of the tea products tested were found to contain high concentrations of Al with large variations observed, ranging from 2.9 to 7.2 mg/L. Among the sub sample of tea products tested the tea product with the highest Al content was found to have the lowest infusible fluoride level. Thus, it is possible that higher amounts of fluoride may be consumed by individuals when the complexity of Al content is taken into consideration. A limitation to this study is that the Al content was not determined in all tea products. Further studies are warranted to determine the concentration and dietary intake of Al and other metal contaminants from tea.

A second limitation is that the study did not take into account the preference of some consumers in the RoI for very strong brewed tea. In the RoI, there is a preference by many consumers to drink tea stronger with more than one tea bag to make a pot of tea. Thus, estimates of fluoride intake from tea in this study are conservative as they are based on a single tea bag to prepare infusions. For consumers who drink strong tea infused in a tea pot prepared with multiple tea bags it is expected that fluoride intake levels would be higher than reported in this study. Tea is also generally consumed with milk in the RoI. However, Gulati *et al.* assessed the effect of adding milk to English style tea and reported that there is no difference between levels of fluoride with or without addition of milk [161]. 

## 5. Conclusions

A number of conclusions can be drawn from this study. First, the main finding of this study is that tea is the major source of exposure to fluoride in the general population. Second, for both adults and children the total dietary intake of fluoride from tea can exceed the upper tolerable intake limit (UL) at levels known to cause chronic fluoride intoxication. Third, while there have been no diagnosed cases of skeletal fluorosis in the RoI we provide evidence to suggest that excessive fluoride may be contributing to the high prevalence of musculoskeletal disorders, undiagnosed clinical stage 1 and stage 2 skeletal fluorosis. This suggests that the lack of any confirmed cases of clinical or sub-clinical fluorosis in the RoI may only reflect a lack of biomonitoring or a lack of awareness of the public health impact of the disease. Fourth, due to the high prevalence of iodine, calcium and vitamin D deficiency, malnutrition and diabetes, we provide evidence to suggest that the high fluoride intake of the population may also be contributing to other disease states. Last, our data suggests the tea products available in the RoI do not comply with EU food law. Taken together, our data and evidence from published literature unequivocally demonstrates that in countries where tea drinking is common fluoridation of public water supplies is unnecessary and potentially harmful. Therefore from a public health perspective, it would seem prudent and sensible that risk reduction measures be implemented to reduce the total body burden of fluoride in the population.

## Figures and Tables

**Table 1:** Tea brands, country of origin and fluoride content in tea infusions.

Sample Number	Brand Name	Origin of Tea	Weight of Tea Bag (g)	Tea Infusions Made with Drinking Water (mg·F/L)	Tea Infusion Made with Deionized Water (mg·F/L)
1	Homestead *	Not listed	3.5	6.1	5.5
2	Tesco Premium ^++^	Not listed	3.4	6.0	5.4
3	Bewleys Original Blend *	KE; RW; a-IN.	3.4	5.5	4.4
4	McGraths Traditional Irish *	Not listed	3.2	5.2	4.6
5	Fallons Irish Blend *	Not listed	3.3	4.8	4.2
6	Barry’s Original Blend *	Not listed	3.8	4.6	3.6
7	Lyons Original Blend ^++^	Not listed	3.6	4.6	3.7
8	Barry's Classic Blend *	Not listed	3.8	4.5	3.3
9	Fallons Original Blend *	Not listed	3.3	4.3	3.8
10	McGraths Decaffeinated	KE.	3.5	4.2	3.2
11	Rob Roberts Organic *	KE; TA.	3.6	4.1	3.2
12	Rob Roberts Kenya Luxury *	KE.	3.6	4.1	3.2
13	Knightsbridge Decaffeinated *	Not listed	3.2	4.1	3.3
14	Daily Basics *	Not listed	2.9	4.0	3.0
15	Fallons Reserve Blend *	Not listed	3.4	3.9	2.9
16	Dunnes Stores Gold Blend *	Not listed	3.3	3.6	2.9
17	Lyons 1 cup ^++^	Not listed	2.6	3.6	2.7
18	Tetley ^++^	Not listed	3.4	3.6	2.7
19	Lipton Yellow label ^++^	Not listed	2.1	3.5	2.6
20	Barry's Decaffeinated *	Not listed	3.4	3.4	2.6
21	Tesco Decaffeinated ^++^	Not listed	3.5	3.4	2.6
23	Barry's Earl Grey *	Not listed	3.0	3.4	2.7
22	Supervalue Gold Blend *	Not listed	3.5	3.3	2.6
24	Lyons Gold Blend ^++^	Not listed	3.5	3.3	2.2
25	Fairglobe Fairtrade *	Africa	2.4	3.3	2.5
26	Rob Roberts Strong Blend *	KE; a-ID	3.4	3.3	2.3
27	Typhoo Irish Breakfast Blend^++^	Not listed	3.2	3.2	2.6
28	Fallons Gold Blend *	Not listed	3.7	3.2	2.5
29	Barry's Gold Blend *	Not listed	3.4	3.1	2.5
30	Clipper ^++^	IN; SL; EA	3.5	3.1	2.4
31	Barber Daly *	Not listed	3.3	3.1	2.5
32	Diplomat Fairtrade *	Not listed	3.3	3.1	2.4
33	Knightsbridge English Breakfast *	Not listed	2.6	2.9	2.4
34	McGraths Gold Blend *	Not listed	3.5	2.9	2.1
35	Sainsbury Basic Fairtrade ^++^	Not listed	2.5	2.9	2.2
36	Typhoo Tea ^++^	Not listed	3.3	2.9	2.0
37	McGraths Reserve Blend *	Not listed	3.5	2.8	1.9
38	Twinings English Breakfast ^++^	Not listed	2.7	2.7	2.1
39	AHMAD tea ^++^	Not listed	2.2	2.7	2.0
40	Typhoo Extra Strong Tea ^++^	Not listed	3.1	2.7	1.9
41	Lyons Decaffeinated ^++^	Not listed	3.5	2.6	2.0
42	Supervalue Original Blend *	Not listed	3.3	2.6	2.1
43	Clipper Organic ^++^	IN; EA;SL	3.3	2.6	1.9
44	Dragonfly Organic Earl Grey	Not listed	2.8	2.6	1.9
45	Bewleys Gold Blend *	Not listed	3.5	2.5	1.7
46	Ridgewood Organic ^++^	Not listed	3.3	2.3	1.6
47	Yorkshire tea ^++^	IN; SL; EA	2.7	2.3	1.7
48	Thompsons Punjana tea ^++^	IN	3.3	2.3	1.5
49	Supervalue Fairtrade tea *	EA	3.4	2.2	1.6
50	Tesco Everyday tea ^++^	Not listed	3.0	2.1	1.5
51	Tesco Finest ^++^	IN; SL; EA	3.3	2.1	1.5
52	Knightsbridge Assam *	a-IN	2.7	2.1	1.4
53	Clipper Organic Assam ^++^	a-IN	2.7	2.0	1.3
54	Clipper Assam Organic Decaf ^++^	a-IN	3.1	1.6	0.8
			***mean***	3.3	2.6

* Irish Blend; ^++^ UK Blend. Three tea bags selected at random from each branded box of tea. Each tea bag was brewed for a period of 5 min with 200 mL of water initially at 100 °C. After five minutes of extraction, tea bags were removed and the infusions were cooled to room temperature (18–20 °C). Fluoride level of boiled and cooled tap water 0.7 mg/L. Fluoride level of deionized water was 0.0 mg/L. The fluoride content of tea infusions made with drinking water are the mean of two samples tested from each brand of tea. Abbreviations: KE = Kenya; TA= Tasmania; RW = Rwanda; EA = East Africa; IN= India; a-IN = Assam India; SL = Sri Lanka.

**Table 2:** Reported “Adequate Intake” (AI) of fluoride for infants, children and adults.

	AI Fluoride from All Sources (mg/day)	
**Age Range**	**Males**	**Females**
0–6 months	0.01 ^†^	0.01 ^†^
7–11 months	0.4 ^‡^	0.4 ^‡^
1–3 years	0.6 ^‡^	0.6 ^‡^
4–6 years	1 ^‡^	0.9 ^‡^
7–10 years	1.5 ^‡^	1.4 ^‡^
11–14 years	2.2 ^‡^	2.3 ^‡^
15–17 years	3.2 ^‡^	2.8 ^‡^
>18 years	3.4 ^‡^; 4 ^†^	2.9 ^‡^; 3 ^†^

**^†^** Institute of Medicine (US) Standing Committee on the Scientific Evaluation of Dietary Reference Intakes. [115]; ^‡^ European Food Safety Authority [9].

**Table 3:** Summary of fluoride intake from tea for infants and children as a percentage of AI and UL based on EU reference levels.

Age-Dependent Risk	150 mL Mean (Range)	250 mL Mean (Range)	750 mL Mean (Range)
Mean intake (mg/day) Range (mg/day)	0.5 (0.2–0.9)	0.83 (0.4–1.5)	2.5 (1.2–4.5)
*Mean (%) fluoride of AI*			
Infants 7–11 months	124 (58–227)	207 (97–378)	621 (291–1134)
Infants 1–3 years	80 (40–150)	138 (65–252)	414 (194–796)
Males 4–6 years		83 (49–151)	248 (116–454)
Females 4–6 years		92 (43–168)	276 (129–504)
Males 7–10 years		55 (26–101)	166 (78–303)
Females 7–10 years		59 (28–108)	177 (83–324)
Females 11–14 years		38 (17–66)	108 (51–197)
Males 11–14 years		36 (18–69)	113 (53–206)
*Mean (%) of UL*			
Infants 0–6 months *	71 (33–130)	118 (55–216)	
Infants 4–12 months *	55 (26–101)	92 (43–168)	
Children 1–3 Years	33 (16–61)	55 (26–101)	166 (78–303)
Children 4–8 Years		33 (16–61)	99 (47–182)
Children 9–14 years		16.5 (7–30)	50 (23–91)

* No UL has been provided for infants under 12 months in the EU by the EFSA. Thus, IOM dietary reference values are.

**Table 4:** Summary of fluoride intake from tea for adults as a percentage of AI and UL based on EU dietary reference.

Risk Characterization	250 mL Mean (Range)	1.0 L Mean (Range)	1.5 L Mean (Range)	3.8 L Mean (Range)
Mean intake (mg/day) Range (mg/day)	0.83 (0.4–1.5)	3.3 (1.6–6.1)	5.0 (2.3–9.1)	12.5 (6.0–23)
**Mean (%) of AI**				
Adult female	28 (13–52)	114 (53–209)	171 (80–313)	432 (203–793)
Adult male	24 (11–44)	97 (46–178)	146 (68–267)	368 (173–676)
**Mean (%) of UL**				
Adult both sexes	12 (5–22)	47 (22–86)	71 (33–130)	179 (84–324)

Values expressed as mean of all tea products and upper and lower range.

## Abbreviations

The following abbreviations are used in this manuscript:

AI: Adequate Intake
EU: European Union
EFSA: European Food Safety Authority
FSAI: Food Safety Authority of Ireland
IQ: Intelligence quotient
NRC: National Research Council
RoI: Republic of Ireland
RME: Reasonable Maximum Exposure
SCHER: Scientific Committee on Health and Environmental Risks
TISAB: Total Ionic Strength Adjustment Buffer
UK: United Kingdom
UL: Tolerable Upper Intake Level
U.S: United States of America
WHO: World Health Organization

## References

[1] Karademir S., Mustafa A., Kuybulu A.E., Olgar Ş., Öktem F. (2011). Effects of fluorosis on QT dispersion, heart rate variability and echocardiographic parameters in children. Anadolu Kardiyol Derg..

[2] National Research Council (2006). Review of Fluoride in Drinking Water, U.S..

[3] Weinstein L.H., Davison A.W. (2004). Fluorides in the Environment, Effects on Plants and Animals.

[4] World Health Organization (1984). Fluorine and Fluoride, Environmental Health Criteria 36.

[5] Reid E. (1936). The fluorine content of some Chinese food materials. Chin. J. Physiol..

[6] Lockwood H.C. (1937). Fluorine in food products. Analyst.

[7] Tokalıoğlu S., Kartal S., Sahin U. (2004). Determination of fluoride in various samples and some infusions using a fluoride selective electrode. Turk. J. Chem..

[8] Rao G.S. (1984). Dietary Intake and Bioavailability of Fluoride. Annu. Rev. Nutr..

[9] European Food Safety Authority (2013). Scientific Opinion on Dietary Reference Values for fluoride, EFSA Panel on Dietetic Products, Nutrition, and Allergies. EFSA J..

[10] Gershon-Cohen J., McClendon J.F. (1957). The cariostatic effect of fluorine in tea. J. Albert Einstein Med. Cent..

[11] Buzalaf M.A., Pessan J.P., Honorio H.M., Cate J.M. (2011). Mechanisms of action of fluoride for caries control. Monogr. Oral Sci..

[12] Water Fluoridation for the Prevention of Dental Caries (Review). http://www.cochrane.org/CD010856/ORAL_water-fluoridation-prevent-tooth-decay.

[13] Scientific Committee on Health and Environmental Risks Critical Review of Any New Evidence on the Hazard Profile, Health Effects, and Human Exposure to Fluoride and the Fluoridating Agents of Drinking Water. http://ec.europa.eu/health/scientific_committees/environmental_risks/docs/scher_o_139.pdf.

[14] Gupta P., Sandesh N. (2012). Estimation of fluoride concentration in tea infusions, prepared from different forms of tea, commercially available in Mathura city. J. Int. Soc. Prev. Comm. Dent..

[15] Krishnamachari K.A. (1986). Skeletal fluorosis in humans: A review of recent progress in the understanding of the disease. Progr. Food Nutr. Sci..

[16] Suttie J W., Phillips P.H., Miller R.F. (1958). Studies of the effects of Dietary Sodium Fluoride on Dairy Cows. J. Nutr..

[17] European Food Safety Authority (2005). Opinion of the Panel on Dietetic Products, Nutrition, and Allergies (NDA) on the tolerable upper intake level of fluoride. EFSA J..

[18] Amalraj A., Pius A. (2013). Health risk from fluoride exposure of a population in selected areas of Tamil Nadu South India. Food Sci. Hum. Wellness.

[19] DenBesten P., Zhu L., Li W., Tanimoto K., Liu H., Witkowska H.E. (2011). Fluoride incorporation into apatite crystals delays amelogenin hydrolysis. Eur. J. Oral Sci..

[20] Gupta S.K., Gambhir S., Mithal A., Das B.K. (1993). Skeletal scintigraphic findings in endemic skeletal fluorosis. Nucl. Med. Commun..

[21] Mostafaei F., McNeill F.E., Chettle D.R., Wainman B.C., Pidruczny A.E., Prestwich W.V. (2015). Measurements of fluorine in contemporary urban Canadians: A comparison of the levels found in human bone using *in vivo* and *ex vivo* neutron activation analysis. Physiol. Meas..

[22] Takamori T., Miyanaga S., Kawahara H., Okushi D., Hirao M., Wakatsuki H. (1956). Electrocardiographic studies of the inhabitants in high fluoride districts. Tokushima. J. Exp. Med..

[23] Wang G.Q., Huang Y.Z., Xiao B.Y., Qian X.C., Yao H., Hu Y. (1997). Toxicity From Water Containing Arsenic and Fluoride in Xinjiang. Fluoride.

[24] Zhou Q.H., Zhang D.C. (1988). Electrocardiogram analysis of 271 dental fluorosis cases. Chin. J. Endemiol..

[25] Xu R.Y., Xu R.Q. (1997). Electrocardiogram analysis of patients with skeletal fluorosis. Fluoride.

[26] Varol E., Akcay S., Ersoy I.H., Ozaydin M., Koroglu B.K., Varol S. (2010). Aortic elasticity is impaired in patients with endemic fluorosis. Biol. Trace Elem. Res..

[27] Varol E., Akcay S., Ersoy I.H., Koroglu B.K., Varol S. (2010). Impact of chronic fluorosis on left ventricular diastolic and global functions. Sci. Total Environ..

[28] Varol E., Varol S. (2012). Effect of fluoride toxicity on cardiovascular systems: Role of oxidative stress. Arch. Toxicol..

[29] Varol E., Varol S. (2013). Water-Borne Fluoride and Primary Hypertension. Fluoride.

[30] Lin F.F., Ai H.T., Zhao H.X., Lin J., Jhiang J.Y. (1991). High fluoride and low iodine environment and subclinical cretinism in Xinjiang. Endemic Dis. Bull..

[31] Xu Y.L., Lu C.S., Zhang X.N. (1994). Effect of fluoride on children’s intelligence. Endem. Dis. Bull..

[32] Seraj B., Shahrabi M., Falahzade M., Falahzade F.P., Akhondi N. (2006). Effect of high fluoride concentration in drinking water on children’s intelligence. J. Dental Med..

[33] Poureslami H.R., Horri A., Atash R. (2011). High fluoride exposure in drinking water: Effect on children’s IQ, one new report. Int. J. Pediatr. Dent..

[34] Lu Y., Sun Z.R., Wu L.N., Wang X., Lu W., Liu S.S. (2000). Effect of high-fluoride water on intelligence in children. Fluoride.

[35] Ding Y., Gao Y., Sun H., Han H., Wang W., Ji X. (2011). The relationships between low levels of urine fluoride on children’s intelligence, dental fluorosis in endemic fluorosis area in Hulunbuir, Inner Mongolia, China. J. Harzard Mat..

[36] Choi A.L., Sun G., Zhang Y., Grandjean P. (2012). Developmental Fluoride Neurotoxicity: A Systematic Review and Meta-Analysis. Environ. Health Perspect..

[37] Zhang S., Zhang X., Liu H., Qu W., Guan Z., Zeng Q. (2015). Modifying Effect of COMT Gene Polymorphism and a Predictive Role for Proteomics Analysis in Children’s Intelligence in Endemic Fluorosis Area in Tianjin, China. Toxicol. Sci..

[38] Peckham S., Lowery D., Spencer S. (2015). Are fluoride levels in drinking water associated with hypothyroidism prevalence in England? A large observational study of GP practice data and fluoride levels in drinking water. J. Epidemiol. Community Health.

[39] Bahijri S., Al-Fares A., Al-Khateeb T., Mufti A. (2006). Sub-Clinical, Undetected Hyperparathyroidism and Hypothyroidism in Individuals Consuming High Fluoride Intake in Jeddah-Saudi Arabia, Implications to Health and Economy. Osteoporos. Int..

[40] Bahijri S.M., Al-Fares A., Al-Khateeb T., Mufti A.B. (2008). Hyperparathyroidism and Hypothyroidism in Individuals Consuming High Fluoride Intake in Jeddah-Saudi Arabia. Syrian Clin. Lab. Assoc..

[41] Xiong X., Liu J., He W., Xia T., He P., Chen X., Yang K., Wang A. (2007). Dose-effect relationship between drinking water fluoride levels and damage to liver and kidney functions in children. Environ. Res..

[42] Liu J.L., Xia T., Yu Y.Y., Sun X.Z., Zhu Q., He W., Zhang M., Wang A. (2005). The dose-effect relationship of water fluoride levels and renal damage in children. Wei Sheng Yan Jiu.

[43] Lung S.C.C., Hsiao P.K., Chiang K.M. (2003). Fluoride concentrations in three types of commercially packed tea drinks in Taiwan. J. Expos. Anal. Environ. Epidem..

[44] Cao J., Luo S.F., Liu J.W., Li Y. (2004). Safety evaluation on fluoride content in black tea. Food Chem..

[45] Cao J., Zhao Y., Li Y., Deng H.J., Yi J., Liu J.W. (2006). Fluoride levels in various black tea commodities: Measurement and safety evaluation. Food Chem. Toxicol..

[46] Lung S.C.C., Cheng H.W., Fu C.B. (2008). Potential exposure and risk of fluoride intakes from tea drinks produced in Taiwan. J. Exposure Sci. Environ. Epidem..

[47] Malinowska E., Inkielewicz I., Czarnowski W., Szefer P. (2008). Assessment of fluoride concentration and daily intake by human from tea and herbal infusions. Food Chem. Toxicol..

[48] Pehrsson P.R., Patterson K.Y., Perry C.R. (2011). The fluoride content of select brewed and microwave-brewed black teas in the United States. J. Food Compos. Anal..

[49] Koblar A., Tavčar G., Ponikvar-Svet M. (2012). Fluoride in teas of different types and forms and the exposure of humans to fluoride with tea and diet. Food Chem..

[50] Quock R.L., Gao J.X., Chan J.T. (2012). Tea fluoride concentration and the pediatric patient. Food Chemistry..

[51] Chan L., Mehra A., Saikat S., Lynch P. (2013). Human exposure assessment of fluoride from tea (*Camellia sinensis* L.). Food Res. Int..

[52] Cao J., Zhao Y., Liu J. (1997). Brick tea consumption as the cause of dental fluorosis among children from Mongol, Kazak and Yugu populations in China. Food Chem. Toxicol..

[53] Bilbeissi M.W., Fraysse C., Mitre D., Kerebel L.M., Kerebel B. (1988). Dental fluorosis in relation to tea drinking in Jordan. Fluoride.

[54] Wang L.F. (1993). Fluorosis and the tea drinking habit among Kazaks in Xinjiang. Chin. J. End. Dis. Bull..

[55] Abuhaloob L., Abed Y. (2013). Dietary behaviours and dental fluorosis among Gaza Strip children. EMHJ.

[56] Cao J., Liu J., Tang L., Ren L. (2005). Early stage skeletal fluorosis in children induced by brick tea fluoride. Chin. J. Endemiol..

[57] Cook H.A. (1971). Fluoride studies in a patient with arthritis. Lancet.

[58] Cao J., Bai X., Zhao Y., Liu J., Zhou D., Fang S. (1996). The relationship of fluorosis and brick tea drinking in Chinese Tibetans. Environ. Health Perspect..

[59] Hayem G., Ballard M., Palazzo E., Somogyi N., Roux F., Meyer O. (2004). Insufficiency bone fractures due to fluorosis in heavy tea drinkers. Ann. Rheum. Dis..

[60] Hallanger-Johnson J.E., Kearns A.E., Doran P.M., Khoo T.K., Wermers R.A. (2007). Fluoride-related bone disease associated with habitual tea consumption. Mayo Clin. Proc..

[61] Whyte M.P., Totty W.G., Lim V.T., Whitford G.M. (2008). Skeletal Fluorosis from Instant Tea. J. Bone Miner. Res..

[62] Izuora K., Twombly J.G., Whitford G.M., Dermertiz J., Pacifici R., Whyte M.P. (2011). Skeletal fluorosis from brewed tea. J. Clin. Endocrin. Metab..

[63] Yi J., Cao J. (2008). Tea and fluorosis. J. Fluorine Chem..

[64] Li H., Liu Q., Wang W., Yang L., Li Y.H., Feng F.J. (2009). Fluoride in drinking water, brick tea infusion and human urine in two counties in Inner Mongolia. Chin. J. Hazard. Mat..

[65] Whyte M.P., Essmyer K., Gannon F.H., Reinus W.R. (2005). Skeletal fluorosis and instant tea. Am. J. Med..

[66] Isbel T.S., Villareal-Armamento R. (2010). What Is Your Guess? A Case of Thick but Brittle Bones and Instant Tea. Clin. Chem..

[67] Joshi S., Hlaing T., Whitford G.M., Compston J.E. (2011). Skeletal fluorosis due to excessive tea and toothpaste consumption. Osteoporos. Int..

[68] Kakumanu N., Rao S.D. (2013). Skeletal Fluorosis Due to Excessive Tea Drinking. N. Engl. J. Med..

[69] Liu X., Wang S., Wang G. Investigation Report of Tea Drinking Fluorosis in Chenbaerhu Flag. http://en.cnki.com.cn/Article_en/CJFDTOTAL-ZDFB200105019.htm.

[70] Experts’ Group of the Ministry of Public Health for Epidemiological survey of fluorosis of Tea drinking Type Fluorosis Institute Harbin Medical Uninersity, Harbin 150096, Epidemiolgical Survey on Skeletal Fluorosis of Drinking Brick-Tea Type in Aba County of Sichuan Province And Chenbaerhuqi County of The Inner Mongolia Autonomous Region. http://en.cnki.com.cn/Article_en/CJFDTOTAL-DYBF200003000.htm.

[71] Liu Q., Wang W., Wang G. (2008). Study on brick tea type fluoride-aluminum combined toxicosis in HuLunBeiEr Inner Mongolia. Chin. J. Control Endem. Dis..

[72] Wang Z., Li S., Liu Q., Liu X. (2011). Investigation on toxicosis of fluorine in Inner Mongolia Agricultural areas. Chin. J. Dis. Mon. Control.

[73] Yang X., Chen J., Den J. (2009). Sichuan Provincial Center for Disease Control and Prevention, Chengdu 610041,China;Epidemioligcal Features of Skeletal Fluorosis by Tea Drinking and Related Factors in Sichuan. Chin. J. Prev. Med. Inf..

[74] Arlaud J., Lam-my S., Desch G., Pierre F. (1984). Osteomalacia disclosing bone fluorosis caused by regular consumption of Vichy Saint-Yorre mineral water. Presse Med..

[75] Del Olmo J.A., Sanmartí R., Alba R., Navasa M.A. (1985). Fluorosis caused by mineral water. Med. Clin. (Barc).

[76] Mazarrasa Mowinckel O. (1986). Fluorosis caused by mineral water in Spain. Med. Clin. (Barc).

[77] Noël C., Gosselin B., Dracon M., Pagniez D., Lemaguer D., Lemaître L. (1985). Risk of bone disease as a result of fluoride intake in chronic renal insufficiency. Nephrologie.

[78] Meunier P.J., Femenias M., Duboeuf F., Chapuy M.C., Delmas P.D. (1989). Increased vertebral bone density in heavy drinkers of mineral water rich in fluoride. Lancet.

[79] Welsch M., Bloch J.G., Stephan D., Bloch R., Imbs J.L. (1990). Iatrogenic fluorosis: 2 Cases. Therapie.

[80] Bottenberg P. (2004). Fluoride content of mineral waters on the Belgian market and a case report of fluorosis induced by mineral water use. Eur. J. Pediatr..

[81] Boulétreau P.H., Bost M., Fontanges E., Lauverjat M., Gutknecht C., Ecochard R., Delmas P.D., Chambrier C. (2006). Fluoride exposure and bone status in patients with chronic intestinal failure who are receiving home parenteral nutrition. Am. J. Clin. Nutr..

[82] Skiles J.L., Imel E.A., Christenson J.C., Bell J.E., Hulbert M.L. (2011). Fluorosis Because of Prolonged Voriconazole Therapy in a Teenager With Acute Myelogenous Leukemia. JCO.

[83] Gerber B., Guggenberger R., Fasler D., Nair G., Manz M.G., Stussi G., Schanz U. (2012). Reversible skeletal disease and high fluoride serum levels in hematologic patients receiving voriconazole. Blood.

[84] Meunier P.J., Courpron P., Smoller J.S., Briancon D. (1980). Niflumic acid-induced skeletal fluorosis: Iatrogenic disease or therapeutic perspective for osteoporosis?. Clin. Orthop. Relat. Res..

[85] Thompson G.R., Baysa D., Cohen S.H., Pappagianisa D. (2012). Fluoride Excess in Coccidioidomycosis Patients Receiving Long-Term Antifungal Therapy: An Assessment of Currently Available Triazoles. Antimicrob. Agents Chemother..

[86] Gras-Champel V., Grados F., Fardellone P., Andréjak M. (2003). Chronic fluorine intoxication during prolonged treatment with niflumic acid. Presse Med..

[87] Kurland E.S., Schulman R.C., Zerwekh J.E., Reinus W.R., Dempster D.W., Whyte M.P. (2007). Recovery from Skeletal Fluorosis (an Enigmatic, American Case). J. Bone Miner Res..

[88] Grennan D.M., Palmer D.G., Malthus R.S., Matagni M.F., de Silva R.T. (1978). Iatrogenic fluorosis. Aust. N. Zeal. J. Med..

[89] Meseg S., Matzkowski H., Hasert V. (1986). Fluorosis following the long-term treatment of osteoporosis with sodium fluoride. Z. Gesamte Inn. Med..

[90] Schmidt C.W., Eisengarten K., Leuschke W. (1986). Therapy-induced fluorosis—Damage or goal?. Z. Gesamte Inn. Med..

[91] Kästner P., Schäfer W. (1988). Skeletal fluorosis following uncontrolled use of sodium fluoride. Z. Arztl. Fortbild.

[92] Franke J. (1988). Fluoride and osteoporosis. Ann. Chir. Gynaecol..

[93] The U.S. Food and Drug Administration (FDA) Regulation 21CFR165.110. http://www.accessdata.fda.gov/scripts/cdrh/cfdocs/cfcfr/cfrsearch.cfm?fr=165.110.

[94] European Commission Directive 2003/40/EC. https://www.fsai.ie/uploadedFiles/Legislation/Food_Legisation_Links/Water/Directive_2003_40_EC.pdf.

[95] Nick H. (2000). The Tea Industry.

[96] Beresniak A., Duru G., Berger G. (2012). Dominique Bremond-Gignac. Relationships between black tea consumption and key health indicators in the world: An ecological study. BMJ Open.

[97] Clarkson L.A., Crawford M.E. (2001). Feast and Famine: Food and Nutrition in Ireland 1500–1920.

[98] (2001). North/South Ireland Food Consumption Survey, Irish Universities Nutrition Alliance.

[99] Willams J., Green S., McNally S., Murray A. (2010). Growing Up in Ireland, A Longitudinal Study of Children.

[100] Freeman V.E. (1996). A Longitudinal Study of Growth, Feeding Practices and Iron Status in Healthy Children from Birth to Age Two Years. Ph.D. Thesis.

[101] Centre for the Promotion of Imports from Developing Countries (CBI), Product Factsheet, Tea in Ireland, 2014. http://www.cbi.eu/sites/default/files/study/product-factsheet-tea-ireland-2014.pdf.

[102] Andersson H.C., Hallstrom H., Kihlman B.A. (2004). Intake of Caffeine and Other Methylxanthines during Pregnancy and Risk for Adverse Effects in Pregnant Women and Their Foetuses.

[103] Tea Statistics Tea Board of India, Kolkatta, 2003. http://www.somo.nl/publications-en/Publication_3092.

[104] Hudaykuliev Y., Tastekin M., Poyrazoglu E.S., Baspinar E., Velioglu Y.S. (2005). Variables affecting fluoride in Turkish black tea. Fluoride.

[105] Giljanovic J., Prkic A., Bralic M., Brkljaca M. (2012). Determination of fluoride in tea infusions by using fluoride ion selective electrode. Int. J. Electrochem. Sci..

[106] Kyle J.A., Morrice P.C., McNeill G., Duthie G.G. (2007). Effects of infusion time and addition of milk on content and absorption of polyphenols from black tea. J. Agric. Food Chem..

[107] (2005). Tea and Coffee Trade Online. http://www.teaandcoffee.net/0305/special.htm.

[108] China Rejects Tea from Kenya over High Fluoride Levels (2015). Business Daily, Kenya.

[109] Kavanagh D., Renehan J. (1998). Fluoride in Tea—Its Dental Significance. J. Ir. Dent. Assoc..

[110] Scientific Committee on Food, Scientific Panel on Dietetic Products, Nutrition and Allergies Tolerable Upper Intake Levels for Vitamins and Minerals, European Food Safety Authority, 2006. http://www.efsa.europa.eu/sites/default/files/efsa_rep/blobserver_assets/ndatolerableuil.pdf.

[111] U.S. Tea Association Fact Sheet: Voluntary Industry Standards for Retail Package Sizes of Tea Bags, Instant Tea, and Iced Tea Mixes. http://www.teausa.org/teausa/images/pos09.pdf.

[112] (2003). Opinion of the Scientific Committee on Cosmetic Products and Non-Food Products Intended for Consumers Concerning The Safety of Fluorine Compounds in Oral Hygiene Products for Children Under The Age of 6 Years. http://ec.europa.eu/health/ph_risk/committees/04_sccp/docs/sccp_o_024.pdf.

[113] Adair S.M., Bowen W.H., Burt B.A., Kumar J.V., Levy S.M., Pendrys D.G. Recommendations for Using Fluoride to Prevent and Control Dental Caries in the United States. http://www.cdc.gov/mmwr/preview/mmwrhtmL/rr5014a1.htm.

[114] Brown T., Mullee A., Collings R., Harvey L., Hooper L., Fairweather-Tait S. Literature Search and Review Related to Specific Preparatory Work in The Establishment of Dietary Reference Values. http://www.efsa.europa.eu/sites/default/files/scientific_output/files/main_documents/283e.pdf.

[115] Institute of Medicine (US) Standing Committee on the Scientific Evaluation of Dietary Reference Intakes (1997). Dietary Reference Intakes for Calcium, Phosphorus, Magnesium, Vitamin D, and Fluoride. http://www.ncbi.nlm.nih.gov/books/NBK109832/.

[116] Scientific Opinion on principles for deriving and applying Dietary Reference Values (2010). EFSA Panel on Dietetic Products, Nutrition, and Allergies. Parma, Italy. EFSA J..

[117] Organisation for Economic Co-operation and Development (OECD) Database on Breastfeeding Rates (CO1.5) 2009. http://www.oecd.org/els/family/43136964.pdf.

[118] Merhav H., Amitai Y., Palti H., Godfrey S. (1985). Tea drinking and microcytic anemia in infants. Am. J. Clin. Nutr..

[119] 22nd Australian Total Diet Study, 2008, Appendix 8. Mean Food Consumption. http://www.foodstandards.gov.au/publications/pages/22ndaustraliantotaldietstudy/Default.aspx.

[120] EFSA NDA Panel (EFSA Panel on Dietetic Products, Nutrition and Allergies) (2015). Scientific Opinion on the safety of caffeine. EFSA J..

[121] Finsterer J. (2002). Earl Grey Intoxication, Case Report. Lancet.

[122] Syed F., Gutierrez A.M., Ghaffar U. (2015). A Case of Iced-Tea Nephropathy. N. Engl. J. Med..

[123] Yuwono M. (2005). Determination of fluoride in black, green and herbal teas by ion selective electrode using a standard-addition method. Maj Ked Gigi.

[124] Kronborg A.I.E. (2013). Trace Elements in Norwegian and Polish Tea Infusions. Master’s Thesis.

[125] USDA National Fluoride Database of Selected Beverages and Foods, Release 2, 2005 December.

[126] Statutory Instrument No 122 of 2014. European Union Drinking Water Regulations 2014.

[127] Chen S., Li B., Lin S., Huang Y., Zhao X., Zhang M. (2013). Change of urinary fluoride and bone metabolism indicators in the endemic fluorosis areas of southern China after supplying low fluoride public water. Public Health.

[128] Székely M., Fazakas Z., Balogh-Sãmãrghiþan V., Bánóczy J. (2010). Urinary Fluoride Excretion after Milk and Tea Consumption in Young Adults. http://oralhealth.ro/volumes/2010/volume-1/V1-10-9.pdf.

[129] Aylward L.L., Hays S.M., Vezina A., Deveau M., St-Amand A., Nong A. (2015). Biomonitoring Equivalents for interpretation of urinary fluoride. Regul. Toxicol. Pharmacol..

[130] Du L., Wan C., Cao X., Liu J. (2008). The Effect of Fluorine on the Developing Human Brain. Translated research report. Fluoride.

[131] Strunecka A., Patocka J., Blaylock R.L., Chinoy N.J. (2007). Fluoride Interactions: From Molecules to Disease. Curr. Signal Transduct. Ther..

[132] Shi J., Dai G., Zhang Z. (1995). Relationship between bone fluoride content, pathological change in bone of aborted fetuses and maternal fluoride level. Zhonghua Yu Fang Yi Xue Za Zhi..

[133] Susheela A.K., Jha M. (1981). Effect of fluoride ingestion on cortical and cancellous bone composition. IRCS J. Med. Sci..

[134] Susheela A.K., Mondal N.K., Singh A. (2013). Exposure to fluoride in smelter workers in a primary aluminum industry in India. Int. J. Occup. Environ. Med..

[135] Weatherell J.A., Smith F.A. (1966). Fluoride and the Skeletal and Dental Tissues. Pharmacology of Fluorides.

[136] Rafferty M.N., Ryan P., Normand C., Murphy A.W., de la Harpe D., McGuire B.E. (2012). The economic cost of chronic noncancer pain in Ireland: Results from the PRIME study, part 2. J. Pain.

[137] Arthritis Ireland. http://www.arthritisireland.ie/go/about_arthritis/arthritis_facts.

[138] Namkaew M., Wiwatanadate P. (2012). Association of fluoride in water for consumption and chronic pain of body parts in residents of San Kamphaeng district, Chiang Mai, Thailand. Trop. Med. Inter Health.

[139] (2012). Institute of Public Health, Musculoskeletal Conditions Briefing. http://www.publichealth.ie/sites/default/files/documents/files/MSC%20Briefing%2004%20Sept%202012.pdf.

[140] Maher B., O’Leary C., Sweeney C., O’Flynn S. (2013). Dietary iodine intake in young Irish women—Cause for concern. Proc. Nutr. Soc..

[141] Nawoor Z., Burns R., Smith D.F., Sheehan S., O’Herlihy C., Smyth P.P.A. (2006). Iodine intake in pregnancy in Ireland—A cause for concern?. Irish J. Med. Sci..

[142] Barry M. Economies in Drug Usage in the Irish Healthcare Setting. http://www.lenus.ie/hse/bitstream/10147/66358/1/economies_drug_usage.pdf.

[143] Ghuran A.V., Camm A.J. (2001). Ischaemic heart disease presenting as arrhythmias. Br. Med. Bull..

[144] Mehta D., Curwin J., Gomes A., Fuster V. (1997). Sudden Death in Coronary Artery Disease: Acute Ischemia *versus* Myocardial Substrate. Circulation.

[145] Department of Health and Children Ireland (2010). Changing Cardiovascular Health National Cardiovascular Health Policy 2010–2019.

[146] Murray M.M., Wilson D.C. (1948). Fluorosis and nutrition in Morocco: Dental studies in relation to environment. Br. Dental J..

[147] Kennedy N. (2011). Irish Society for Parenteral and Enteral Nutrition (ISPEN) Basic Nutrition Support Study Day for SpRs. http://www.irspen.ie/wp-content/uploads/2014/10/IrSPEN_SpR_Nutrition_Study_Day_Malnutrition_In_Context.pdf.

[148] Rice N., Normand C. (2012). The cost associated with disease-related malnutrition in Ireland. Public Health Nutr..

[149] (2013). Update on Osteoporosis. National Medicines Information Centre. http://www.stjames.ie/GPsHealthcareProfessionals/Newsletters/NMICBulletins/NMICBulletins2013/NMIC%20Osteoporosis%20with%20refs.pdf.

[150] Mitchell G.A., Haddad L.M., Winchester J.F. (1983). The management of fluoride poisoning. Clinical Management of Poisoning and Drug Overdose.

[151] (2011). National University of Ireland, Press Release. https://www.nuigalway.ie/about-us/news-and-events/news-archive/2012/november2012/new-diabetes-research-centre-for-the-west-.htmL.

[152] Regulation (EC) No 178/2002 of The European Parliament and of The Council of 28 January 2002 Laying down The General Principles and Requirements of Food Law, Establishing The European Food Safety Authority and Laying down Procedures in Matters of Food Safety. http://eur-lex.europa.eu/legal-content/en/TXT/?uri=CELEX:32002R0178.

[153] CODEX International Food Standards. http://www.codexalimentarius.org/standards/list-of-standards/.

[154] (2013). European Commission. Food safety Risk Analysis—Fifty Years with Codex Alimentarius in the European Region.

[155] World Health Organization (WHO) (1994). Fluorides and Oral Health.

[156] McDonagh M., Whiting P., Bradley M., Cooper J., Sutton A., Chestnutt I., Misso K., Wilson P., Treasure E., Kleijnen J. (2000). Fluoridation of Drinking Water: A Systematic Review of Its Efficacy and Safety.

[157] Cochran J.A., Ketley C.E., Arnadottir I.B., Fernandes B., Koletsi-Kounari H., Oila A.M., Van Loveren C., Whelton H.P., O’Mullane D.M. (2004). A comparison of the prevalence of fluorosis in 8-year-old children from seven European study sites using a standardized methodology. Comm. Dent. Oral Epidem..

[158] World Health Organization (WHO) (2002). Fluorides, Environmental Health Criteria 227.

[159] Colina J.M., Arias C.F., Rodriguez A. (1990). The influence of the composition of the Tisab Solution on the determination of fluoride in tea infusions. Fluoride.

[160] Janiszewska J., Balcerzak M. (2013). Analytical Problems with the Evaluation of Human Exposure to Fluorides from Tea Products. Food Anal. Methods.

[161] Gulati P., Singh V., Gupta M.K., Vaidya V., Dass S., Prakash S. (1993). Studies on the leaching of fluoride in tea infusions. Sci. Total Environ..

